# Exploration of Gancao Xiexin decoction for treatment of Behcet disease based on network pharmacology and molecular docking

**DOI:** 10.1097/MD.0000000000031277

**Published:** 2022-10-21

**Authors:** Xin Zhang

**Affiliations:** Department of Ophthalmology, The First Affiliated Hospital of Zhejiang Chinese Medical University (Zhejiang Provincial Hospital of Traditional Chinese Medicine), Hangzhou, China.

**Keywords:** Behçet disease, Gancao Xiexin decoction, molecular docking, network pharmacology

## Abstract

Based on network pharmacology and molecular docking, this study discusses the potential mechanism of Gancao Xiexin decoction in the treatment of Behçet disease (BD) to provide a more reliable theoretical basis for the treatment of BD.

**Methods:**

The effective components and action targets of Gancao Xiexin decoction were obtained by searching the traditional Chinese medicine systems pharmacology database and analysis platform database, and the genome annotation database platform (GeneCards) database Search BD related targets in Online Mendelian inheritance in man database, pharmacogenetics and pharmacogenomics knowledge base database, therapeutic target database and drugbank database, Venny analysis tool was used to overlap drug targets and disease targets; The “active drug active ingredient target gene” network was constructed with the help of Cytoscape 3.8.2 software, and the protein–protein interaction (PPI) network was constructed with string database; R language was used for Gene Ontology (GO) enrichment analysis and Kyoto Encyclopedia of genes and genomes (KEGG) pathway enrichment analysis; Target prediction based on pubchemp platform.

**Results:**

A total of 163 active components were identified, with 730 corresponding targets, including 56 common targets of the active components and BD. GO enrichment analysis yielded 1126 entries for biological processes (BP), 17 entries for cellular components, and 89 entries for molecular functions. The significant items of BP enrichment mainly included reaction to lipopolysaccharide, reaction to bacteria-derived molecules, exogenous apoptosis signal pathways, and biological metabolism processes of reactive oxygen species. KEGG pathway enrichment analysis identified 118 significantly enriched pathways. The molecular docking technology verified that its effective components can effectively bind to the corresponding BD related target proteins.

**Conclusion:**

Gancao Xiexin decoction synergistically treats BD through multi-component, multi-target, and multi-channel mechanisms, which provides a basis for further study of the active components and mechanism of Gancao Xiexin decoction.

## 1. Introduction

Behçet disease (BD) is a chronic systemic vascular inflammatory disease involving the nervous system, digestive tract, lungs, kidneys, epididymis, and other organs, and is characterized by recurrent oral ulcers, vulvar ulcers, ophthalmia, and skin damage.^[1]^ At present, it is considered to be closely related to genetic, infectious, immune, and environmental factors.^[2]^ There is no effective cure for this disease. The traditional use of drugs, including glucocorticoids, immunosuppressants, non-steroidal drugs, and biological agents, is to control existing symptoms and disease activities, prevent and treat damage to important organs, reduce disease recurrence, and delay the development of the disease.^[3]^ However, BD often cycles from onset to remission and recurrence. The long-term use of hormones and immunosuppressants will inevitably have an impact on the body. Therefore, traditional Chinese medicine methods are sought to further understand the characteristics and curative effects of the disease. There is a discussion on “Fox confusion disease” in Treatise on febrile diseases, which holds that BD is similar. When the heat evil is multiplied, the throat swells and aches, and when wet evil is injected, the two Yin will fester. This can also be seen in Article 10 of the synopsis of the golden chamber. Treatment of Yin-Yang toxin disease and pulse syndrome of lily and fox: “Fox confusion is a disease. Gancao Xiexin decoction is the main”. This disease is characterized by the accumulation of dampness and heat. Dampness blocks heat depression and damages blood collaterals. This disease is based on the accumulation of dampness and heat. Dampness blocks heat depression and heat hurts blood collaterals.^[4]^The “Gancao Xiexin decoction” is based on the theory of the Qi mechanism mediated by Zhongjiao,^[5]^ pay attention to the regulation of the lifting hub of the coke gas machine in the spleen and stomach. In modern clinical application, Gancao Xiexin decoction has significant curative effects on BD,^[6]^ recurrent oral ulcer,^[7]^ ankylosing spondylitis,^[8]^ rheumatoid diseases and other diseases have significant curative effects.

Based on the traditional Chinese medicine systems pharmacology and analysis platform database, this study screened out the main effective components of traditional Chinese medicine in Gancao Xiexin decoction and potential intersection targets with BD and used network pharmacology to explore the mechanism of Banxia Xiexin decoction in the treatment of BD, providing a more reliable theoretical basis for treatment.

## 2. Materials and Methods

### 2.1. Ingredients and targets of traditional Chinese medicine

The main traditional Chinese medicine components of Gancao Xiexin decoction “licorice,” “Scutellaria baicalensis,” “ginseng,” “Coptis chinensis,”“dried ginger,” “jujube” and “Pinellia ternate” were searched through traditional Chinese medicine systems pharmacology and analysis platform database. They were screened according to the criteria of bioavailability (OB) greater than 30% and drug likeness (DL) >0.18. The active components and corresponding targets of seven traditional Chinese medicines were obtained by using the Perl language.

### 2.2. Identify relevant targets of BD

The disease target genes related to BD were retrieved from the GeneCards database, Online Mendelian inheritance in man database, pharmacogenetics and pharmacogenomics knowledge base database, therapeutic target database, and DrugBank database with “Behcet disease” as the keyword. The related genes were input as potential targets for analyzing the active components in Gancao Xiexin decoction in the treatment of BD.

### 2.3. Construction of compound active ingredient, action target network

Using R’s Venndiagram package, the drug disease common target obtained by the above method is drawn into a Venn diagram. The active components in Gancao Xiexin decoction and the action target for the treatment of BD were introduced into Cytoscape 3.8.2 at the same time, and the active component and action target network were constructed.

### 2.4. Construction and analysis of core target interaction network

The potential target genes of the Gancao Xiexin decoction for BD were introduced into the string database. The species was defined as “Homo sapiens,” and the target protein interaction relationship was retrieved and saved in TSV format. The TSV file into Cytoscape 3.8.2 software again, and the network analyzer tool for network analysis was used to obtain the degree value. A protein‐protein interaction (PPI) network was drawn using Cytoscape 3.8.2 software.

### 2.5. Biological process and pathway analysis

Using the dose, clusterprofiler, ggplot2, and pathview packages of R, Gene Ontology (GO) enrichment analysis and metabolic pathway enrichment analysis of Kyoto Encyclopedia of Genes and Genomes (KEGG) were carried out for the component target proteins of Gancao Xiexin decoction. The biological processes (BP) and pathways (*P* < .05) were screened, and the top 20 BP and pathways were selected according to the gene proportion (gene ratio), gene enrichment histogram and bubble diagram were drawn, and pathway information map was obtained to find relevant targets of Gancao Xiexin decoction in the treatment of BD.

### 2.6. Molecular docking technology

By simulating the intermolecular interactions between receptors and ligands, the potential binding mode and affinity between drug molecules and the three-dimensional structure of the target protein were predicted, to screen the effective components and simulate the drug targets. The key target proteins and the main effective components of traditional Chinese medicine were selected based on the degree value of drug components in the drug target disease network diagram, the connection of network adjacent nodes and KEGG enrichment analysis results. The molecular structure of the target protein was obtained from the RCSB PDB protein structure database. After the above molecules were hydrotreated and dewatered with PyMOL, the molecular docking verification of effective components of traditional Chinese medicine and key target proteins was carried out using AutoDock 1.5.6 software, and the docking results were visualized using PyMOL software.

## 3. Results

### 3.1. Active compounds and targets of Gancao Xiexin decoction

Two eighty chemical constituents of licorice, 143 chemical constituents of Scutellaria baicalensis, 190 chemical constituents of ginseng, 148 chemical constituents of dried ginger, 48 chemical constituents of Coptis chinensis, 133 chemical constituents of jujube and 116 chemical constituents of Pinellia ternata were retrieved. Limited by OB ≥ 30.00% and drug class DL ≥ 0.18, 92 qualified active ingredients licorice, 36 Scutellaria baicalensis, 22 ginseng, 5 dried ginger, 14 Coptis chinensis, 29 jujube and 13 Pinellia ternata were selected for data analysis (Table 1).

**Table 1:** Some active components of Gancao Xiexin decoction.

TCM	Active ingredients	OB (%)	DL
Gancao	Glycyrrhizin	90.77	0.66
	7,2’,4’-trihydroxy-5-methoxy-3-arylcoumarin	83.71	0.27
	Licorice coumarin	80.36	0.65
	shinpterocarpin	80.29	0.72
	Phaseol	78.76	0.57
Huangqin	NEOBAICALEIN	104.34	0.43
	Panicolin	76.25	0.29
	5,7,4’-trihydroxy-8-methoxyflavanone	74.23	0.26
	Skullcapflavone II	69.51	0.43
	2,6,2’,4’-tetrahydroxy-6’-methoxychaleone	69.03	0.21
Banxia	(3S,6S)-3-(benzyl)-6-(4-hydroxybenzyl)piperazine-2,5-quinone	46.88	0.26
	beta-D-Ribofuranoside, xanthine-9	44.71	0.20
	Stigmasterol	43.82	0.75
	12,13-epoxy-9-hydroxynonadeca-7,10-dienoic acid	42.15	0.24
	Baicalin	40.12	0.75
Ganjiang	Sexangularetin	62.85	0.29
	[(1S)-3-[(E)-but-2-enyl]-2-methyl-4-oxo-1-cyclopent-2-enyl](1R,3R)-3-[(E)-3-methoxy-2-methyl-3-oxoprop-1-enyl]-2,2-dimethylcyclopropane-1-carboxylate	62.51	0.3
	1-Monolinolein	37.17	0.30
	beta-sitosterol	36.91	0.75
	sitosterol	36.91	0.75
Huanglian	Methyl Berberine	104.95	0.77
	quercetin	86.71	0.26
	Palmitonin A	64.60	0.64
	Luteolin	63.70	0.18
	Berberine	55.36	0.77
Dazao	Spiradine A	113.52	0.60
	Mauritine D	89.12	0.45
	Moupinamide	86.71	0.26
	Ziziphin_qt	66.94	0.61
	Fumarine	59.26	0.82
Ginseng	Celabenzine	101.88	0.48
	Aposiopolamine	66.64	0.21
	Frutinone A	65.90	0.34
	Inermin	65.83	0.53
	Girinimbin	61.21	0.31

DL = drug likeness, OB = bioavailability.

### 3.2. Prediction of related targets of Gancao Xiexin decoction in the treatment of BD

**Figure 1.**
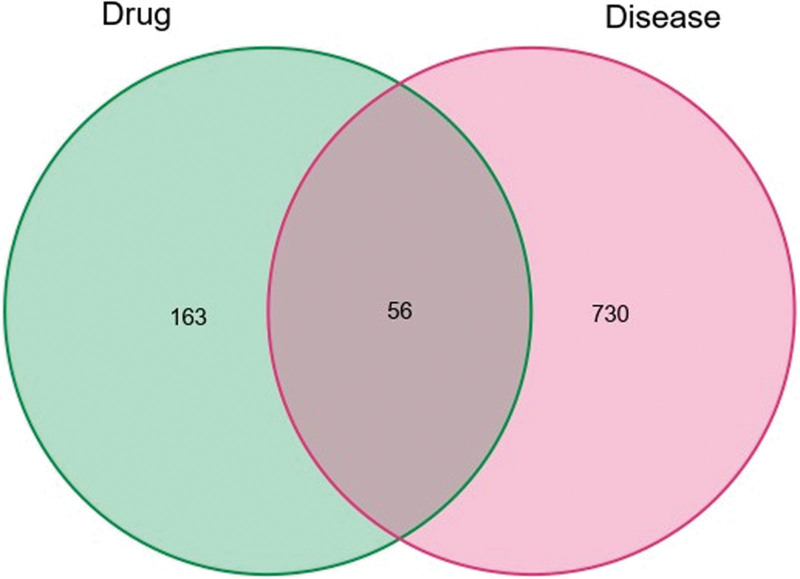
Venn diagram of drug disease common target genes of active ingredient related target genes and BD related genes of Gancao Xiexin decoction. *Note*: The colored circular node represents the active ingredients and different drug flavors, orange and Scutellaria baicalensis; green Coptis chinensis; rose red dried ginger; fluorescein licorice; lake blue jujube; red Pinellia ternata; and dark blue ginseng. The blue rectangle represents the common target. BD = Behçet disease.

The corresponding target proteins were inputted into the UniProt database to obtain the corresponding genes. BD-related genes were compared with the GeneCards, Online Mendelian inheritance in man, pharmacogenetics and pharmacogenomics knowledge base, therapeutic target database, and DrugBank databases. Fifty six common target proteins were identified through the intersection of the drug target and disease-related target proteins. The Venn diagram of the common drug disease target (Fig. 1).

### 3.3. Network diagram of active components of traditional chinese medicine ‐ target protein ‐ disease

**Figure 2.**
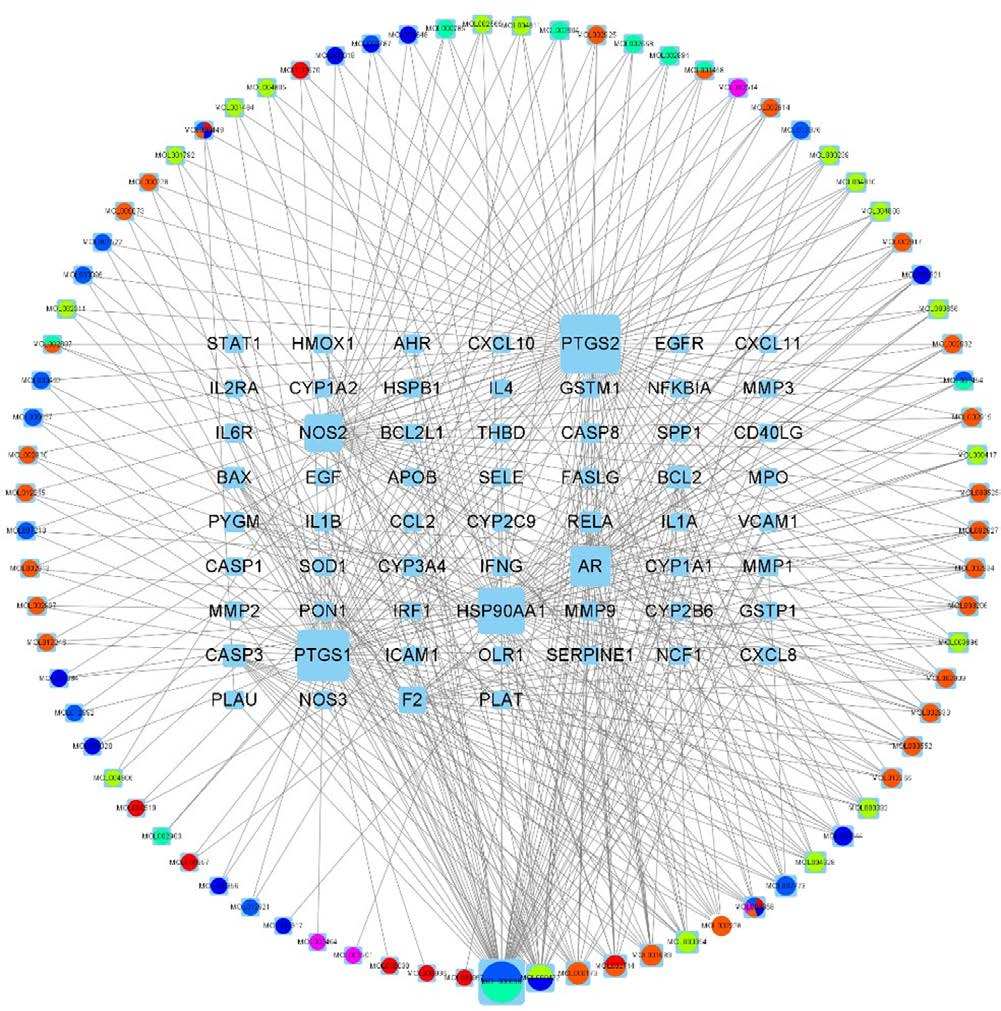
Active ingredient target network of Gancao Xiexin decoction.

The obtained active components and BD-related targets of Gancao Xiexin decoction were introduced into Cytoscape 3.8.2 software to construct the network diagram of active components, target proteins and diseases of traditional Chinese medicine (Fig. 2). The nodes with different colors and shapes in the figure represent the active components the action targets, respectively. The blue rectangular node represents 60 common targets, and the colored ring node represents the active components and different drug flavors. There are multiple links between the drug components and the disease target proteins in the figure, which shows that the Gancao Xiexin decoction is rich in effective components and has multi-target therapeutic effects.

### 3.4. Construction of PPI network diagram of drug disease common target protein

**Figure 3.**
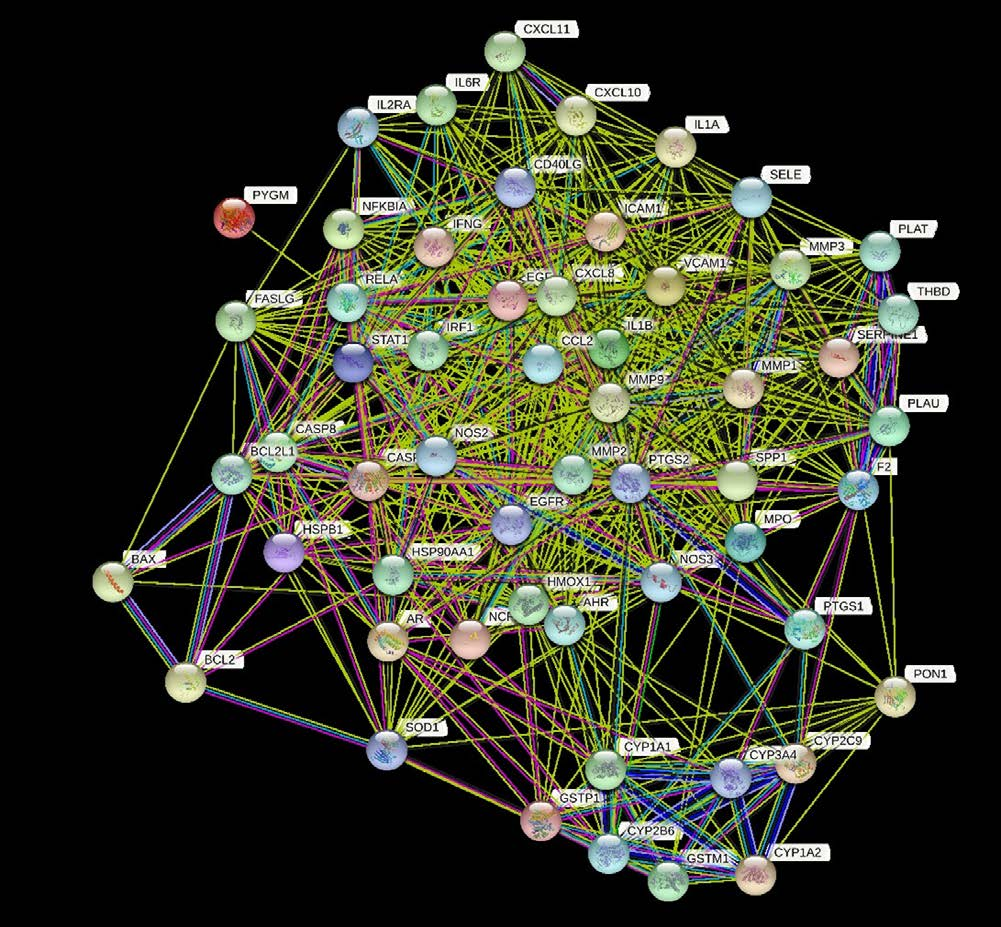
Visualization of Gancao Xiexin decoction BD common target protein PPI. BD = Behçet disease, PPI = protein protein interaction.

**Figure 4.**
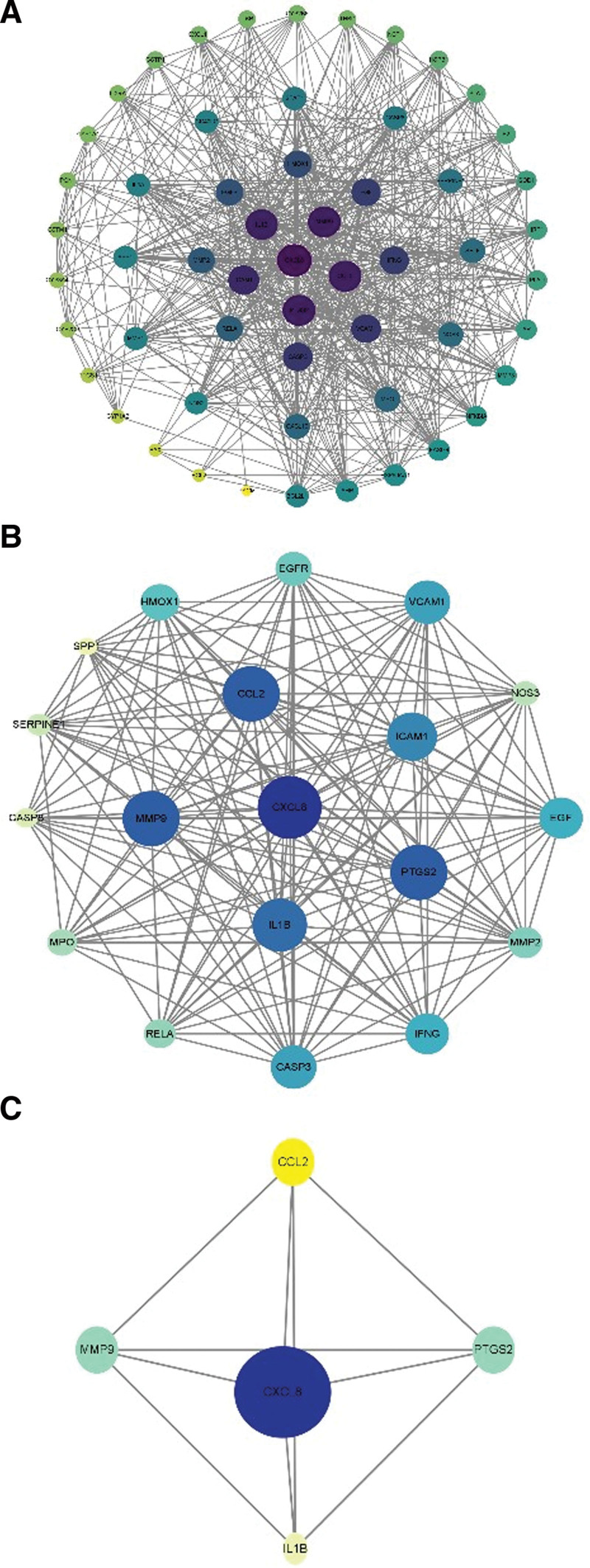
The genes according to the degree value, and the core genes are screened out. (A) First screening, (B) second screening and (C) third screening.

Upload the drug disease common target to the string platform, limit the species to “Homo sapiens,” obtain the protein interaction relationship, and draw the interaction network diagram using Cytoscape 3.8.2 software. The network contains 56 nodes and 640 edges. In the figure, nodes represent proteins and the edges represent the correlation between proteins (Fig. 3). According to the degree value, the genes were filtered twice (Fig. 4A and B), and five core genes were screened, namely interleukin-8 (target gene name: CXCL8), prostaglandin G/H synthase 2 (target gene name: PTGS2), matrix metalloproteinase-9 (target gene name: MMP9), small inducible cytokine A2 (target gene name: CCL2), Interleukin-1 β (target gene name: IL1β) (Fig. 4C).

### 3.5. GO enrichment analysis and KEGG enrichment analysis

**Figure 5.**
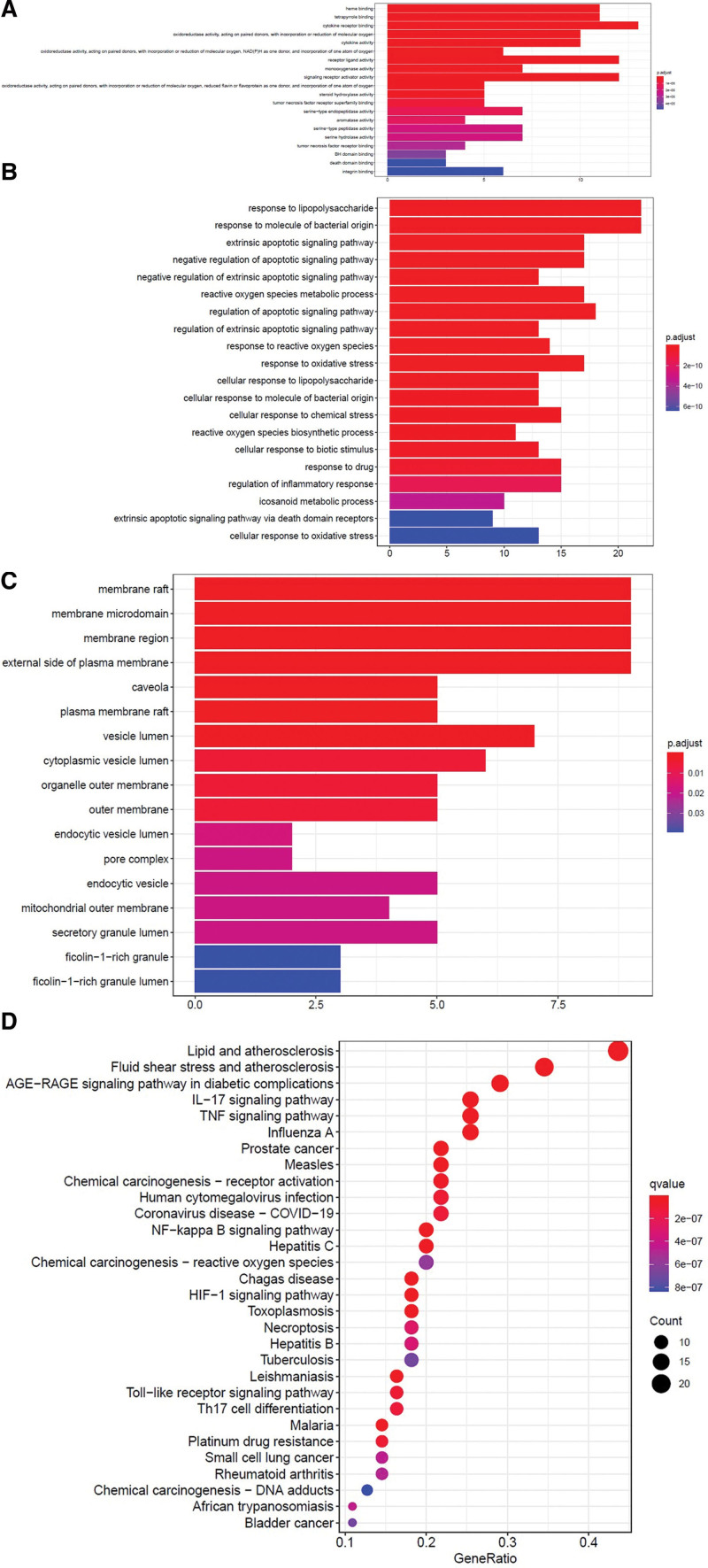
GO enrichment analysis and KEGG enrichment analysis of Gancao Xiexin decoction BD common target: (A) histogram of go gene enrichment molecular function (MF), (B) GO gene enrichment biological process subtype (BP) histogram, (C) GO gene enriched cellular component (CC) histogram and (D) KEGG signaling pathway gene enrichment bubble diagram. BD = Behçet disease, GO = Gene Ontology, KEGG = Kyoto Encyclopedia of Genes and Genomes.

Through the cluster profiler package of R language, GO enrichment analysis of drug disease common targets was carried out, and *P* < .05 was set as the screening condition to obtain 89 molecular function (MF) subtype processes, 1126 biological processes (BP) subtype processes and cellular component (CC) related to the treatment of BD with Gancao Xiexin decoction There are 17 subtype processes. Select the top 20 BP in each subtype according to the degree value, and list the go gene enrichment degree histogram in (Fig. 5A‐D). The length (i.e., ordinate) of the column represents the enrichment degree of the drug targets in the process, and the color of the column represents the adjusted *P*-value. The smaller the valve, the smaller is the *P*-value.

### 3.6. Molecular docking verification results

**Figure 6.**
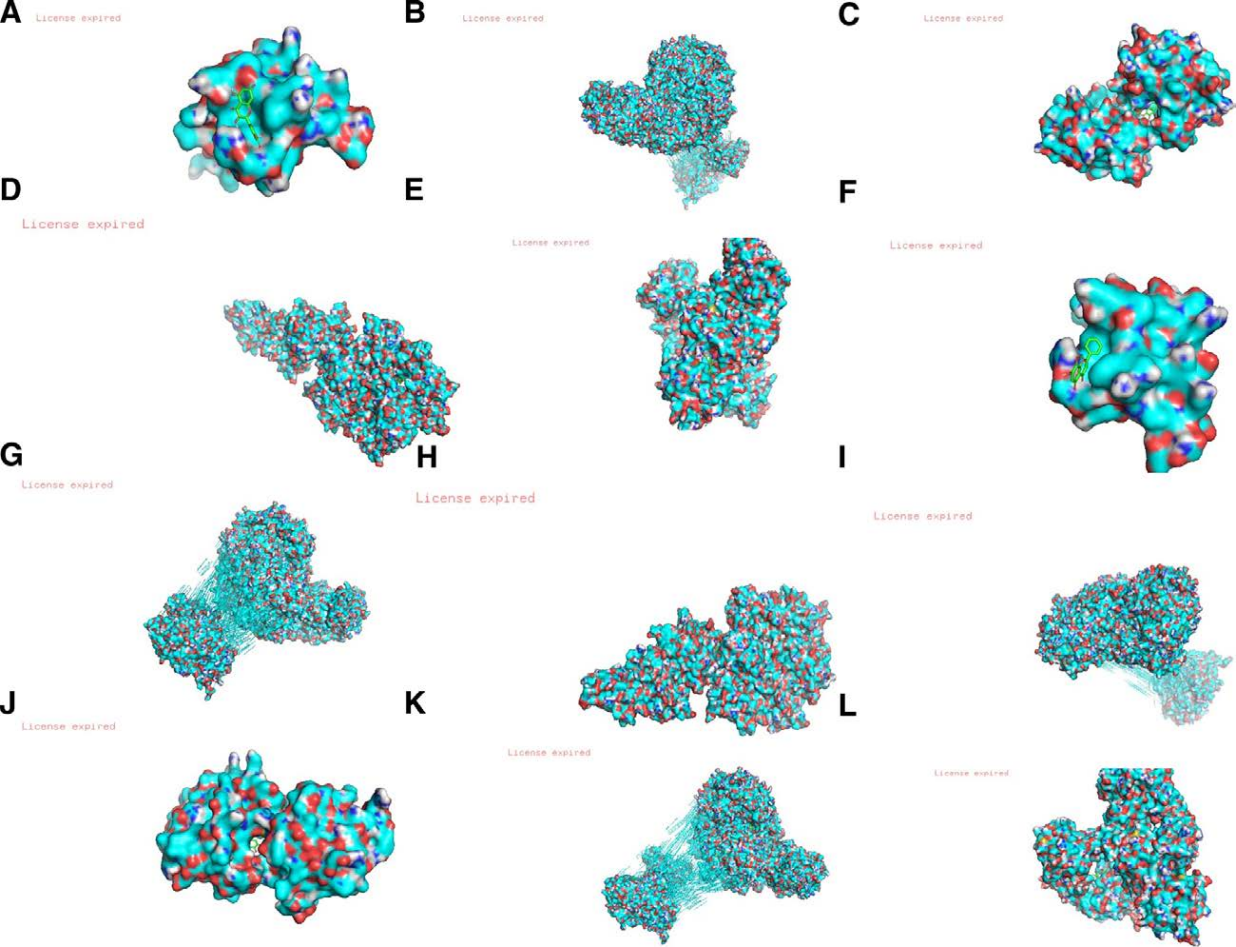
(A‐L) Molecular docking diagram of active components and key targets of Gancao Xiexin decoction.

According to the ranking of degree value in PPI, the core genes were screened using Cytoscape 3.8.2. As receptor proteins, and their related active components, quercetin, baicalein, wogonin and ginsenoside Rh2 were used for molecular docking verification. The molecular docking binding energies and parameters are shown in Table 2 and Figure 6A‐L, respectively. The lower the binding energy, the more stable the binding between the molecule and target protein.

**Table 2:** Scoring and docking parameters of docking binding energy between key compounds and key target molecules.

Protein	Grid size	Docking score (kcal/mol)			
		Quercetin	Wogonin	Baicalein	Ginsenoside rh2
CXCL8	40*40*40	–6.8	–6.8	–	–
PTGS2	40*40*40	–10.4	–10.0	–10.8	–9.7
MMP9	40*40*40	–10.7	–	–10.7	–
CCL2	40*40*40	–8.0	–8.0	–	–
IL1β	60*86*76	–8.0	–	–	–7.7

CCL2 = cytokine A2, CXCL8 = interleukin-8, IL1β = Interleukin-1β, MMP9 = matrix metalloproteinase-9, PTGS2 = prostaglandin G/H synthase 2.

## 4. Discussion

Gancao Xiexin decoction is one of the representative prescriptions for the treatment of PI syndrome of disharmony between the spleen and stomach and mixed cold and heat caused by mistakenly treating typhoid fever. Based on the clinical manifestations of the disease, modern doctors flexibly use the Gancao Xiexin decoction to treat BD. They believe that the medical pathogenesis always belongs to the deficiency of spleen and stomach qi and accumulation of damp heat and evil toxins. He Jun^[9]^ and others applied the Gancao Xiexin decoction to treat BD, and the total effective rate was 86.7%. According to the Golden Book of the medical school, “the prescription named after licorice means moderation.” The sweet temperature of licorice and jujube is used to supplement the middle and urgent, which is beneficial to the treatment of ruffians; the pungent of Pinellia can break the customer’s inverse; the pungent heat of Qin and Lian xie yang can sink the ruffian heat, and the pungent cold of dried ginger can dissipate the Yin coagulation. It is necessary to break the inverse in a hurry and reduce the cold and heat of ruffians.^[4]^ This study integrated bioinformatics and network pharmacology methods to analyze the main bioactive components and pharmacological mechanism of Gancao Xiexin decoction in the treatment of BD. at the same time, molecular docking method was used to verify the binding ability between active components and key targets.

Quercetin, wogonin, baicalein, and ginsenoside are the main active components of the Gancao Xiexin decoction. Quercetin is a flavonoid with chemopreventive and therapeutic effects in a variety of diseases, including inhibition of oxidative stress, cytokines and COX2 production.^[10]^ Previous studies have shown that quercetin can reduce pain and inflammation related to arthritis, and inhibit mechanical hyperalgesia, edema, and leukocyte aggregation of the knee joint in mice in a dose-dependent manner.^[11]^ Its mechanisms include inhibition of proinflammatory cytokines (TNF) in human peripheral blood monocytes-α). The proliferation and gene level of IL-1 are decreased, while the expression of inflammatory factors such as IL-6 and matrix metalloproteinases (MMP3 and MMP9) can inhibit the production of inflammatory mediators and improve inflammatory symptoms. Animal experiments have shown that quercetin can improve pain symptoms by inhibiting the activation of MMP-9 and MMP-2 in microglia in the mouse spinal cord. Levels of interleukin-17 (IL-17) and monocyte chemoattractant protein-1 (MCP-1).^[12]^ Quercetin has antioxidant, free radical scavenging, anti-apoptotic, and immune regulatory functions. Baicalein is an inhibitor, of antibacterial xanthine oxidase, antioxidants, and immune regulation.^[13]^ It can effectively inhibit the itching of free radicals produced during xanthine oxidation. Its hydroxyl structure also has free radical scavenging activity, and through the regulation of the peanut tetraenoic acid metabolic pathway, it plays a powerful antipyretic and analgesic effect by inhibiting the production of cyclooxygenase, dehydrogenase, nuclear factor, and cytokines. Animal experiments have shown that baicalein can inhibit the macrophage inflammatory response induced by lipopolysaccharide and adenosine triphosphate.^[14]^ Ginsenoside Rh2 is a tetracyclic triterpene saponin monomer.^[15]^ In addition to promoting the apoptosis of tumor cells, it also has significant anti-inflammatory effect, which can inhibit the inflammatory response induced by bacterial endotoxin (LPS). Bi^[16]^ reported that rh2-b2 modified with water-soluble groups significantly inhibited the TNF of RAW264.7 macrophages-α, IL-6, IL-1β. In addition, Rh2 can inhibit the production of PGE2, ROS, and MMP-9 in LPS stimulated RAW264.7 macrophages and TNF in keratinocytes-α induced MMP-9 gelatinase activity.

Through the analysis of drug disease action targets, 56 common targets were obtained from the intersection, including 19 main targets and five key targets, namely CXCL8, PTGS2, IL1β, MMP9, and CCL2, and docking them with the active components of Gancao Xiexin decoction. CXCL8 is an inflammatory chemokine that has several of biological functions. Studies have shown that^[17]^ it promotes inflammatory cell drive and plays a key role in the pathogenesis of BD. PTGS2, also known as cyclooxygenase (cox1/2), is a key rate limiting enzyme for the conversion of arachidonic acid to prostaglandins. Meltem believes that^[18]^ the increased activity of (cox1/2) in plasma may be the cause of intravascular inflammation in patients with BD. IL1β is a proinflammatory cytokine, that can continuously promote the release of endogenous arachidonic acid, resulting in the persistent hypersensitivity of mechanical nociceptors, and the increase in inflammatory cytokines is involved in the process of vascular injury caused by BD.^[19]^ MMP-9 plays an important role in immune inflammation, cell migration, proliferation, and apoptosis. It was found that^[20]^ MMP-9 polymorphism is related to BD susceptibility. In the inflammatory state, immune cells adhere to the surface of endothelial cells by secreting inflammatory factors and vascular cell adhesion molecule 1, inducing their expression of MMP-9, and promoting the cross endothelial cell surface of immune inflammatory cells, upregulating of MMP-9 activity. As a chemotactic protein, CCL2 can chemotactic monocytes to specific tissues to eliminate foreign pathogenic microorganisms, produce antibodies and promote the repair of tissue damage; Found its polymorphism in erythrocytes of patients with BD.^[21]^

From the enrichment analysis results, it can be seen that the bioactive components of Gancao Xiexin decoction act on multiple targets to treat BD in a variety of ways, involving a variety of signaling pathways, such as lipid and atherosclerosis, fluid shear stress, atherosclerosis, AGE-RAGE signal pathway, TNF signal pathway, and IL-17 signaling pathway. Studies have shown that the AGE-RAGE pathway can participate in the regression of inflammation, maintenance of intracellular homeostasis, and repair and regeneration after injury and that TNF-α mediates cell necrosis and apoptosis in a variety of ways and plays an important role in the occurrence and development of BD.

In summary, this study applied network pharmacology to screen the bioactive components of Gancao Xiexin decoction and its effective action targets in BD, which reflects that the Gancao Xiexin decoction has the characteristics of multi-target and multi-channel treatment, provides ideas for the follow-up study of its action mechanism in the treatment of BD, and provides new scientific connotations to traditional Chinese medicine compounds at a new level. In addition, more clinical trials and animal trials are needed for further verification.

## Author contributions

**Conception and design of study:** ZX.

Data collection: ZX.

Drafting the manuscript: ZX.

The author reviewed the results and approved the final version of the manuscript.
